# Modulation of Fear and Arousal Behavior by Serotonin Transporter (5-HTT) Genotypes in Newly Hatched Chickens

**DOI:** 10.3389/fnbeh.2018.00284

**Published:** 2018-11-20

**Authors:** Valerie D. Phi Van, E. Tobias Krause, Loc Phi-Van

**Affiliations:** ^1^Institute of Diagnostic and Interventional Radiology, University Hospital Zürich, Zürich, Switzerland; ^2^Institute of Animal Welfare and Animal Husbandry, Friedrich-Loeffler-Institut (FLI), Celle, Germany

**Keywords:** serotonin transporter, 5-HTT, 5-HTT polymorphism, fear, early post-hatching periods, *Gallus gallus*

## Abstract

The serotonin transporter (5-HTT) plays a key role in regulating serotonergic transmission via removal of serotonin (5-hydroxytryptamine, 5-HT) from synaptic clefts. Alterations in 5-HTT expression and 5-HT transmission have been shown to cause changes to adult behavior including fear. The objective of the present study was to investigate the 5-HTT role in fear in birds at the very early stages of post-hatching life. Using an avoidance test with an elevated balance beam, which was based on depth perception and the respective fear of heights, we assessed fear-related avoidance behaviors of newly hatched chicks of the three functional 5-HTT genotypes W/W, W/D and D/D. Newly hatched chicks of the genotype D/D, which was linked to high 5-HTT expression, showed less intensive avoidance responses as measured by decreased latency to jump than W/W and W/D chicks. Further, significantly fewer D/D hens than W/W hens showed fear-like behavior that resembled a freezing response. Furthermore, in an arousal test the arousal reaction of the chicks in response to an acute short-term visual social deprivation in the home compartment was assessed 5 weeks after hatching, which also revealed that D/D chicks exhibited decreased arousal reaction, compared to W/W chicks. Thus, the results indicate that fear responses differ in D/D chicks in the early post-hatching periods, possibly due to the different expression of 5-HTT respectively 5-HT levels in this strain.

## Introduction

Neuronal serotonin (5-hydroxytryptamine, 5-HT) is widely distributed in the central nervous system and has been shown to be a major mediator of several physiological and neurobehavioral processes, including locomotion (Holmes et al., 2003; Uçeyler et al., 2010), feeding (Lam et al., 2010), emotion (Jonassen and Landrø, 2014), impulsivity (Landrø et al., 2015; Nomura et al., 2015) and fear-related behavior (Gross and Hen, 2004; Berger et al., 2009). The 5-HT transmission and downstream postsynaptic responses are controlled via reuptake of 5-HT from synaptic clefts by the serotonin transporter (5-HTT; Blakely et al., 1994). The human 5-HTT gene has a number of polymorphisms which may influence the 5-HTT expression. The most well-known example is the polymorphism with a 44-bp insertion/deletion mutation in a 5-HTT promoter region (5-HTT-linked polymorphic region, 5-HTTLPR) which causes long (*l*) and short (*s*) alleles and strongly correlates with anxiety traits in patients (Lesch et al., 1996). It was shown that human lymphoblast cells carrying two copies of the *s* allele had reduced 5-HTT gene expression, leading to a decrease in 5-HT uptake when compared to cells with one or two copies of the *l* allele (Lesch et al., 1996). Further, individuals carrying two copies of the *s* allele showed significantly lower 5-HTT expression in the brain than individuals with *l/l* and *s/l* genotypes, which are clinically linked with depression and varying response to alcohol abuse (Little et al., 1998; Heinz et al., 2000; Pezawas et al., 2005). The amygdala, a part of the limbic system and responsible for the processing of emotions, especially fear responses, seems to be critical for mediating 5-HT effects on fear-related behaviors in mammals (Bocchio et al., 2016). In birds, the arcopallium/posterior pallial amygdala is considered homologous to the mammalian amygdala. Behavioral studies following lesions damaging the arcopallium indicated that this brain region is involved in the control of fear-related behaviors (Phillips, 1964; Cohen, 1975; Dafters, 1975; Phillips and Youngren, 1986; Saint-Dizier et al., 2009).

It was reported that *s/l* and *s/s* carriers, as fear increased, exhibited greater amygdala neuronal activity in blood oxygen level-dependent functional magnetic resonance imaging (fMRI) than individuals with two copies of the *l* allele (Hariri et al., 2002). Similar mechanisms for 5-HTT in regulating 5-HT effects on fear-related behaviors seems to be available also in the amygdalar homologous arcopallium in birds (Krause et al., 2017). Moreover, several human gene association studies have provided increasing evidence indicating that *s/s* carriers exhibited increased risk for depression, decreased stress resistance and increased fear-related traits compared with carriers of *s/l* and *l/l* genotypes (Lesch et al., 1996; Caspi et al., 2003; Karg et al., 2011). In line with these findings, studies using knockout mice demonstrated that mice deficient of 5-HTT exhibited more fear-related traits and impaired fear extinction recall than wild-type mice (Holmes et al., 2003; Wellman et al., 2007), while other studies with 5-HTT-overexpressing mice demonstrated reduced brain 5-HT levels and low-fear-related behavior (Jennings et al., 2006; Barkus et al., 2014; Bocchio et al., 2015).

Increasing evidence indicates the role of 5-HT on the development of neuronal circuits during the post-natal life in rodents (Daubert and Condron, 2010; Teissier et al., 2017). High 5-HT brain levels in 5-HTT knock-out mice or in rats exposed to the 5-HT selective reuptake inhibitor fluoxetine during the post-natal development could hinder the maturation of the 5-HT raphe neurons (Lira et al., 2003; Silva et al., 2010). Further, it was found that the thickness of layer IV is decreased in 5-HTT knock-out mice compared with wild-type mice (Altamura et al., 2007) and similarly reduced gray matter brain volumes are associated also with the 5-HTTLPR *s* allele (Frodl et al., 2008). In particular, several studies intensively focused on the post-natal effect of 5-HT on the formation of cortical microcircuits in the somatosensory cortex, the so-called barrel-cortex in rodents (Daubert and Condron, 2010; Teissier et al., 2017). Increasing 5-HT in monoamine oxidase A knock-out mice and 5-HTT knock-out rats as well as in rats exposed to fluoxetine resulted in a reduction of the thalamocortical axon (TCA) terminal branches and morphological changes in dendritic organization (Rebsam et al., 2002; Lee et al., 2009; Miceli et al., 2013). Also, altered dendritic morphologies in pyramidal neurons in the infralimbic cortex and basolateral amygdala that are associated with responses to emotional stimuli were found in 5-HTT knock-out mice (Wellman et al., 2007). All the genetic 5-HTT alterations in humans and mice mentioned above have been shown to affect emotionality in adulthood (Daubert and Condron, 2010). However, it is still unclear whether the behavioral phenotypes in adulthood might be due to acute changes in 5-HT concentrations or rather due to altered neuronal circuit development. Pharmacological studies have shown that administration of fluoxetine or 5-methoxytryptamine, a 5-HT agonist, in the early post-natal periods altered emotional behaviors in adulthood, providing indirect evidence for the role of altered neuronal circuits (Ansorge et al., 2004; Dennis et al., 2013a).

Previously, we reported a functional polymorphism in the 5′-flanking region of the chicken 5-HTT gene. The D variant is caused by a deletion of four nucleotides (5′-AATT-3′) and a closely spaced single nucleotide substitution (A→T) compared to the wild-type variant W. In contrast to the human *s* allele, which reduces the 5-HTT expression, the chicken deleted D allele was found to increase the 5-HTT expression compared to the wild-type W allele (Phi-van et al., 2014). Moreover, the D allele was shown to be associated with increased locomotion (Phi-van et al., 2014) as well as with increased feed intake and growth (Kjaer and Phi-van, 2016). Recently, we found that adult hens carrying the D allele showed lower levels of fear than hens with the W allele (Krause et al., 2017). Because all the altered behavioral behaviors have been found in adult animals, we further examined the role of the 5-HTT in fear-like behavior (i.e., fear-related avoidance and social arousal behavior when visually deprived from conspecifics) in newly hatched birds and in young birds in the present study. Especially, using an avoidance test we addressed the question of whether the 5-HTT genotypes could affect the fear behaviors in chicks at the very early stages of post-hatching life.

## Materials and Methods

### Animals

Chicks (*Gallus gallus domesticus*) were raised from 5-HTT W/W and D/D parents (Phi-van et al., 2014). Briefly, 20 cocks of each genotype were randomly intercrossed with 20 hens of the same genotype and with 10 hens of the other genotype. After 21 days of incubation, all newly hatched chicks were collected from the incubator, sorted (only females were considered here) and individually marked with numbered wing tags. Immediately afterwards, 219 females kept in three standard brooding rings (73 chicks of all three genotypes per ring) with infrared brooding lamps in a large compartment were tested in the avoidance and righting response tests. The chicks had continuous visual, acoustic and olfactory contact to the conspecifics during the tests. Both tests were conducted with the same chicks (77 W/W, 69 W/D and 73 D/D) at an age of less than 48 h post hatching. To randomize and minimize potential age-related differences in behavior and motoric capabilities, all chicks were tested in random order on the same day (9 am to 1 pm). After testing, 219 chicks of the three genotypes were housed in the same compartment (6.5 × 3.6 m), which floor was equipped with litter. The light was provided for 14 h per day and temperature maintained constantly at 18°C. Chicks had unlimited access to food and water. In the third behavioral test, the arousal test, a reduced number of the same chicks (71 W/W, 60 W/D and 65 D/D) were tested on the open field in this home compartment at 5 weeks of age. Thus, for none of the three behavioral tests chicks were removed from the home compartment. This study was carried out in accordance with the guidelines of the European Communities Council Directive of 22 September 2010 (2010/63/EU) and the German Animal Protection Law. The animal testing was approved by the local authorities of Lower Saxony.

### 5-HTT Genotyping

Genomic DNA was isolated from 3 μl whole blood using a GeneJET Genomic DNA Purification kit from Fermentas (St. Leon-Rot, Germany) according to the instructions of the manufacturer. To amplify the polymorphic 5-HTT region, PCR was performed with 0.5 μg genomic DNA using specific primers (281, 5′-CGCTCGCAGCACAAAGGAT-3′ and 282, 5′-GACAAAGCTTGACCCCCATAC-3′) as described previously (Phi-van et al., 2014). For 5-HTT genotyping, 1 μl of each PCR sample was digested in 15 μl of standard buffer with 10 units of the restriction endonuclease Vsp I or Mun I for 3 h at 37°C. The digested DNA fragments were then resolved on 2% agarose gels containing 1× TBE (89 mM Tris-HCl, 89 mM boric acid, 2 mM EDTA, pH 8.3). After electrophoresis, gels were stained with 0.5 μg/ml ethidium bromide in 1× TBE and photographed under UV-illumination. Alternately, the PCR samples were directly analyzed by polyacrylamide gel electrophoresis for 5-HTT genotyping as described previously (Phi-van et al., 2014).

### Avoidance Test

The test was conducted using an elevated narrow balance beam (6 cm wide and 35 cm long). The balance beam was 22 cm high, so that chicks could jump down onto soft bedding without injuring themselves. The balance beam aimed at representing a novel situation (height and depth) for newly hatched chicks. Thus, we expected using this test to assess fear-related behaviors, particularly fear of heights and the respective avoidance response, similar to tests in rodents (Pellow et al., 1985). Chicks were tested once only. The test was performed as follows. Each chick was placed on the starting line at an end of the balance beam and then allowed to walk. We recorded the following parameters: (i) the latency for the chick to jump off the beam; (ii) the distance the chick walked on the beam; and (iii) the overall occurrence of specific behaviors (e.g., failure to jump) in the three different groups. If a chick stayed on the starting line or was unable to walk on the beam, it received a score of 0. The test lasted a maximum of 2 min. If a chick failed to jump off the beam within 2 min, its time was recorded as 120 s.

### Righting Response Test

To assess the vestibular functions and basic motor coordination, the righting response test was performed by placing the chick on its back and then immediately allowing it to return to a standing position. Thus, the test significantly differs from the tonic immobility (TI) test which is commonly used to measure fear in chickens. In the TI test, the TI is induced by restraining the chick on its back for at least 10 s before the duration of immobility is measured (Gallup et al., 1971; Jones, 1986). This step was omitted in the righting response test. The righting response of each chick was observed and recorded using a Sony HDR-CX220E video camera recording 25 frames per second. Afterwards, the time chicks required to right was calculated using Video deluxe 17 Premium (MAGIX, Berlin, Germany) with an accuracy of 40 ms. If a chick was unable to right itself within 3 s twice, it received a maximum score of 3 s i.e., 3,000 ms.

### Arousal Test

In the arousal test, the arousal reaction to a brief visual separation from the group of conspecifics was examined, i.e., chicks were tested for 3 min and subsequently immediately released to the peer-group. The test arena was surrounded by a 0.5-m high wall to visually separate focal birds from conspecifics. But acoustic and olfactory contact between the separated chick and the flock remained possible. The arena was located in the home compartment. The test is from the set-up similar to an open field test, but with important modifications, i.e., that the test arena was located within the home compartment and the separated chick had full acoustic and olfactory contact to the conspecifics. The test arena consisted of 25 fields (5 × 5), each measuring 20 × 20 cm. For arousal testing, each chick was taken from the flock and immediately placed in the center of the arena in full acoustic contact to the conspecifics (Vallortigara, 1988), and the locomotor activity was measured. As locomotor activity we counted the total number of movements between fields in the arena with 25 (5 × 5) fields, in response to the brief visual separation. A higher locomotor activity reflects a higher arousal. Similar tests have been shown to test for arousal in several animal taxa (Archer, 1973; but see Ennaceur and Chazot, 2016) including chicken, where this arousal also has a social component (Gallup and Suarez, 1980; Faure et al., 1983; Heiblum et al., 1998). Especially, the presence of conspecific calling has important impact on the behavior in such tests, regardless of the nature of the conspecific calls, i.e., distress calls or trills of pleasure (Vallortigara, 1988; Vallortigara and Zanforlin, 1988).

### Statistical Analysis

Kruskal-Wallis test with Dunn’s multiple comparisons correction was performed on behavioral data which were not normally distributed and could not be transformed to a normal distribution. Chi-square and Fisher’s exact tests were used to calculate categorical data. Normally distributed data were analyzed with one-way ANOVA followed by Tukey HSD *post hoc* test. Statistical analysis was performed using GraphPad Prism 7 (GraphPad Software, San Diego, CA, USA), and *P* < 0.05 was considered statistically significant.

## Results

### Restriction Enzymes for 5-HTT Genotyping

**Figure 1:**
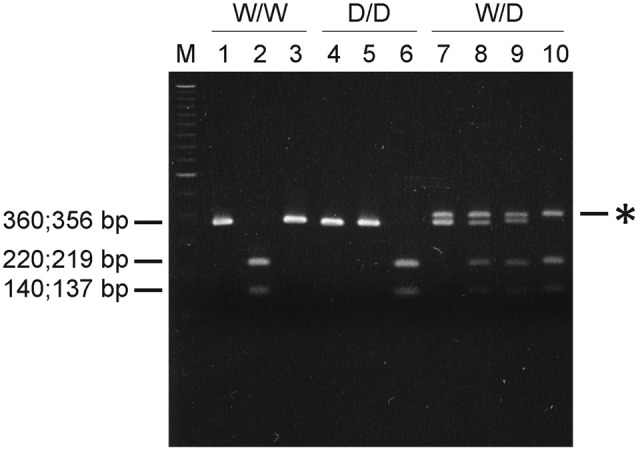
Serotonin transporter (5-HTT) genotyping using restriction endonucleases. PCR was performed with 0.5 μg genomic DNA from W/W, W/D or D/D chicks and DNA primers 281 and 282. PCR products were digested without (lanes 1, 4 and 7) or with restriction endonucleases Vsp I (lanes 2, 5 and 8) or Mun I (lanes 3, 6 and 9) or with both enzymes (lane 10). Digested DNA fragments were then resolved by electrophoresis on a 2% agarose gel, stained with ethidium bromide and visualized under UV-illumination. Asterisk (*) indicates the ~0.4-kb heteroduplex formed from W and D. DNA markers used were the 100-bp ladder (lane M).

Vsp I digests the sequence ATTAAT, which is present in the wild type W variant (5′-CAATTAATTG-3′), whereas Mun I recognizes the sequence CAATTG present in D variant lacking the four nucleotides 5′-AATT-3′. Figure 1 demonstrates the results obtained in a representative experiment. The PCR fragment of 360 bp in length from genomic DNA of a W/W homozygote was digested with Vsp I, but not with Mun I, into two sub-fragments (140 bp and 220 bp), and in contrast, the 356-bp PCR fragment from genomic DNA of a D/D homozygote was cleaved with Mun I, but not with Vsp I, into two sub-fragments (137 bp and 219 bp). PCR of genomic DNA isolated from a W/D heterozygote resulted in two DNA fragments (356 and 360 bp) and a heteroduplex of ~0.4 kb detected on the agarose gel. The 356- and 360-bp DNA fragments were completely digested with Mun I and Vsp I, respectively, whereas the ~0.4-kb heteroduplex, which was artificially formed by hybridization of W and D during the last step of PCR, was resistant to Vsp I and Mun I.

### Evaluation of Genotype Effects on Fear-Related Avoidance Behaviors

**Figure 2:**
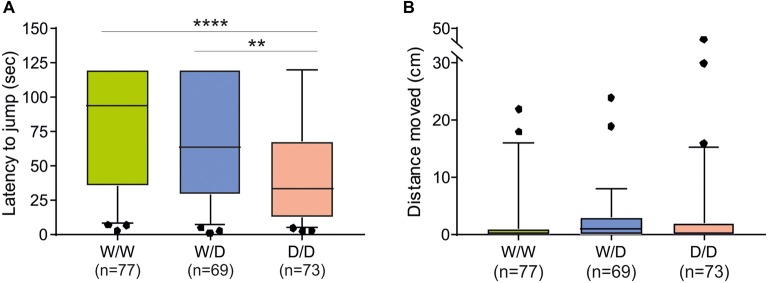
Behavioral performance of newly hatched chicks of three genotypes (W/W, W/D and D/D) in the avoidance test. **(A)** The latency to jump off differed between chicks of the three genotypes (*P* < 0.0001). **(B)** The distance moved on the balance beam was not significantly different between chicks of the three genotypes. Box plots show medians, first and third quartiles and whiskers with outliers outside the 5th and 95th percentiles. **P < 0.01, ****P < 0.0001.

The avoidance test with the elevated balance beam revealed differences between chicks of the three 5-HTT genotypes. The latency for chicks to jump off the beam differed significantly between 5-HTT wild type W/W, heterozygous W/D and homozygous D/D chicks: W/W chicks spent the longest time and D/D spent the shortest time on the beam and W/D chicks were intermediate (Kruskal-Wallis test: *N*_W/W_ = 77, *N*_W/D_ = 69, *N*_D/D_ = 73; *χ*^2^ = 20.27, *df* = 2, *P* < 0.0001; Figure 2A). *Post hoc* comparisons revealed significant differences between W/W and D/D (*P* < 0.0001) and between W/D and D/D (*P* = 0.0067), but no significant difference between W/W and W/D (*P* > 0.05). However, the distance chicks moved on the beam was very short and not significantly different between chicks of the three genotypes (Kruskal-Wallis test: *N*_W/W_ = 77, *N*_W/D_ = 69, *N*_D/D_ = 73; *χ*^2^ = 3.89, *df* = 2, *P* = 0.14; Figure 2B).

Monitoring the reaction type of chicks revealed that they behaved differently after being placed on the beam, e.g., the majority of chicks left the beam by jumping off, but there were many chicks which failed to walk and to jump off. Therefore, we examined whether the 5-HTT genotypes affected these behaviors of chicks. Table 1 shows numbers of chicks of the three genotypes showing different behaviors on the balance beam. There were 3.5 times more W/W chicks than D/D chicks, which failed to walk and jump off the beam, and in contrast, 1.8 times more D/D chicks than W/W chicks, which failed to walk but jumped off the beam. Analysis using the Chi-square test revealed a significant relationship between the genotypes and these behaviors of chicks on the balance beam (*χ*^2^ = 18.46, *df* = 6, *P* = 0.0052). Both walking and jumping behavior of chicks are separately obtained after being placed on the beam to investigate irrespectively of each other. For walking behavior, only walking and no-walking chicks were considered. The numbers of chicks which walked or did not walk on the beam were not significantly different between the three genotypes (*χ*^2^ = 2.81, *df* = 2, *P* = 0.24). Thus, there was no interaction between walking behavior and the genotypes. In contrast, Chi square analysis of the numbers of chicks which jumped off or did not jump off the beam, irrespective of the walking behavior, revealed that the homozygous D/D genotype was significantly less frequent among birds which failed to jump off (*χ*^2^ = 10.35, *df* = 2, *P* = 0.0056; Table 2). Pairwise comparisons showed that there was a significant difference between W/W and D/D genotypes (Fisher’s exact test, *P* = 0.0019). Next, to assess a possible association of the W allele and D allele with jumping behavior, W and D allele frequencies in 155 jumping chicks and 64 no-jumping chicks were calculated and analyzed using Fisher’s exact test (Table 2). Chicks which failed to jump off carried the D allele significantly less frequently than chicks which were able to jump off (*P* = 0.0002). Thus, the W and D alleles were significantly associated with no jumping and jumping, respectively.

**Table 1:** Distribution of the serotonin transporter (5-HTT) genotypes in chicks exhibiting walking and jumping behaviors in the avoidance test.

		Genotypes		
Subjects	*N*	W/W	W/D	D/D
nW-nJ	47	26	14	7
nW-J	69	19	17	33
W-nJ	17	5	7	5
W-J	86	27	31	28

*nW-nJ, chicks failed to walk and to jump off; nW-J, chicks failed to walk, but jumped off; W-nJ, chicks walked, but failed to jump off; W-J, chicks walked and jumped off. Chi-square analysis: *χ*^2^ = 18.46, df = 6, P = 0.0052*.

**Table 2:** Interaction between the alleles W and D and jumping behavior of chicks in the avoidance test.

		Genotypes			Alleles	
Subjects	*N*	W/W	W/D	D/D	W	D
J	155	46	48	61	140	170
nJ	64	31	21	12	83	45

*J, chicks jumped off irrespective of walking behavior; nJ, chicks failed to jump off irrespective of walking behavior. Genotypes: Chi-square analysis, *χ*^2^ = 10.35, df = 2, P = 0.0056. Alleles: Fisher’s exact test, P = 0.0002*.

### Evaluation of Genotype Effects on the Vestibular and Motoric Related Righting Response

**Figure 3:**
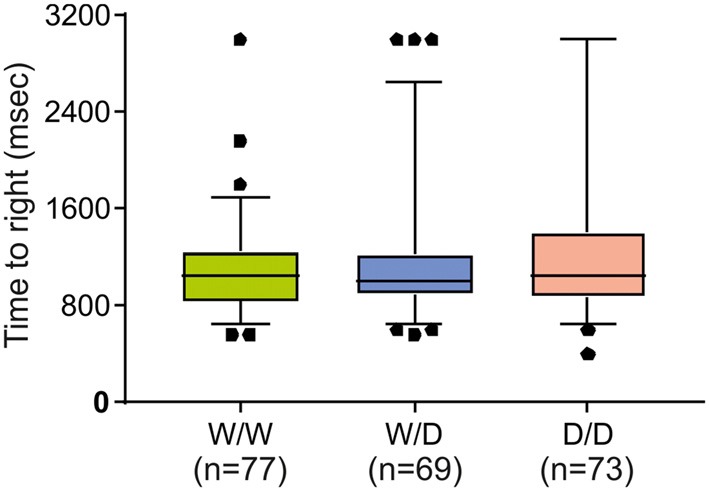
Righting response of newly hatched chicks of three genotypes (W/W, W/D and D/D). The time required to right was not significantly different between chicks of the three genotypes. Box plots show medians, first and third quartiles and whiskers with outliers outside the 5th and 95th percentiles.

Further, to examine the question of whether the worse performance of W/W chicks in the avoidance test might be due to possible impairments in vestibular or motor functioning, the righting response test was performed. As shown in Figure 3, righting responses did not significantly differ between newly hatched chicks of the three genotypes (Kruskal-Wallis test: *N*_W/W_ = 77, *N*_W/D_ = 69, *N*_D/D_ = 73; *χ*^2^ = 0.26, *df* = 2, *P* = 0.88).

### Evaluation of Genotype Effects on Social Deprivation-Induced Arousal Behaviors

**Figure 4:**
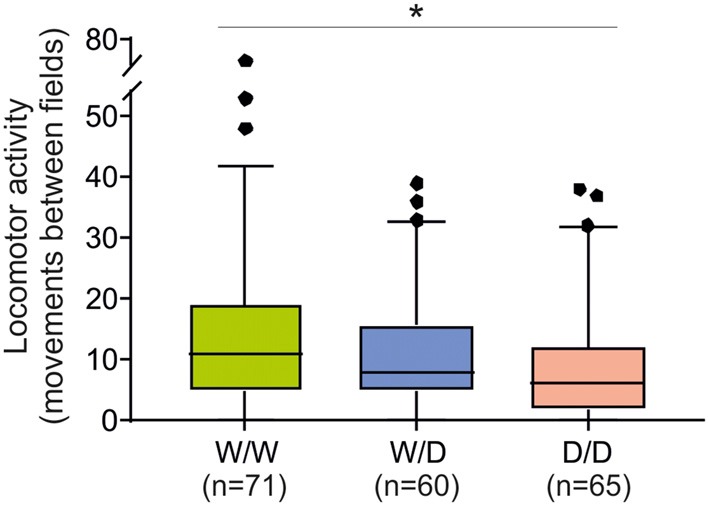
Locomotor activity of 5-week old chicks of three genotypes (W/W, W/D and D/D) in the open field test. The total number of movements between fields was different between W/W and D/D chicks (*P* = 0.015). Box plots show medians, first and third quartiles and whiskers with outliers outside the 5th and 95th percentiles. *P < 0.05.

The total number of movements between fields as an indicator of locomotor arousal activity reaction was significantly different between chicks of the three genotypes (Kruskal-Wallis test: *N*_W/W_ = 71, *N*_W/D_ = 60, *N*_D/D_ = 65; *χ*^2^ = 7.93, *df* = 2, *P* = 0.019). W/W chicks moved between fields more often than W/D and D/D chicks (Figure 4), indicating a higher arousal of W/W chicks. The *post hoc* pairwise comparisons revealed that the difference in the number of movements between fields between W/W and D/D chicks was significant (*P* = 0.015) and the difference between W/W and W/D chicks and between W/D and D/D chicks were not significant (both *P* > 0.05).

## Discussion

Our results indicate that chicks with the 5-HTT polymorphism at early post-hatch ages and at 5 weeks of age showed significantly different levels of fear and arousal. These differences were linked to the three genotypes in response to behavioral tasks in the avoidance test or an arousal reaction following the brief visual separation from conspecifics, respectively. Further, the results suggest that the W allele, which is linked to low 5-HTT expression, is associated with higher fear response, while the D allele, which is linked to high 5-HTT expression, is associated with lower fear response.

Like rodents, birds seem to have a neuronal 5-HT system which is present but probably not fully mature at early post-hatch ages (Daubert and Condron, 2010; Dennis et al., 2013a,b; Teissier et al., 2017). In the context of the 5-HT role in the development of neural circuits controlling emotion during the early post-hatch life, our results showing altered fear-related behaviors by 5-HTT genotypes in chicks at early post-hatch ages might suggest that these behavioral alterations might be due at least in part to acute changes in 5-HT levels.

The avoidance test with an elevated balance beam is based on one of the most fundamental biologically adaptive emotions, the fear of heights and the respective avoidance response in humans as well as in earthly animals including chicken (Gibson and Walk, 1960). Using a visual cliff, Walk and Gibson (Walk and Gibson, 1961) showed that the depth perception and thus the respective fear of heights are already developed in chicks at an age less than 24 h after hatching, and when tested on the visual cliff, all the newly hatched chicks avoided the deep side of 10 inch. Further, Walk et al. (1968) demonstrated that chicks showed first a preference for the shallow side when the depth of the cliff was more than 2 inches. These findings indicated that emotions including fear and avoidance were significant for the choice of the shallow side. Testing the avoidance behavior, our simple test apparatus, a narrow balance beam, has a critical height of 22 cm, which allowed chicks to jump off the beam without injury. On the other hand, this apparatus represented obviously a great challenge for chicks expressing more intensively their levels of fear. First, the latency to jump off the beam was significantly longer in W/W chicks than in D/D chicks (Figure 2A), and second, there were many chicks which, after being placed on the starting line, did not move a step or sat down, i.e., they failed/avoided to walk on and to jump off the beam within 120 s (26 of 77 W/W chicks vs. 7 of 73 D/D chicks; Table 1). This behavior resembled an initial freezing response described by Schaller and Emlen (1962) and Phillips and Siegel (1966). The authors demonstrated that initial fear response of newly hatched chicks to several visual or auditory stimuli was an inhibition of peeping and other ongoing activity, which then led frequently to crouching and freezing, and silent freezing often passed directly over into sleep. Thus, our results might provide factual circumstances sufficient to conclude that the latency for chicks to jump off the beam reflect different levels of fear: more fearful W/W chicks spent a longest time while less fearful D/D chicks spent a shortest time.

Another aspect of avoidance testing with the balance beam is the social separation, when chicks were taken from the group for immediate testing. Indeed, several studies demonstrated that when faced with the threat of social isolation, chicks began to peep and then moved about the test arena in order to reinstate social contact with the conspecifics (Gallup and Suarez, 1980; Vallortigara, 1988; Vallortigara and Zanforlin, 1988). If the same is true for chicks in the avoidance test and 5-HTT genotypes would affect social behaviors, one would expect that W/W and D/D chicks differ in motivation for social reinstatement. However, this presumption cannot be in line with the following considerations: first, that the 5-HTT is implicated in controlling social behaviors as already shown in mammals (Bethea et al., 2004; Homberg et al., 2007), yet remains unclear in chickens. Second, the most important point is that the avoidance test was performed on the same day newly hatched chicks were collected from the incubator, i.e., they were very little reared socially prior to testing. Indeed, studies using 2-day old chicks showed that the presence of a companion or conspecific calls had no effects on the ambulation latency in the open field test, suggesting that very young chicks possibly have very little social experience (Vallortigara, 1988). Third, nevertheless, to minimize the effects of social isolation, the avoidance test was performed within the home compartment, i.e., the chicks had continuous visual, acoustic and olfactory contact to the conspecifics. The presence of familiar and imprinted companions was shown to reduce stress (Wilson, 2000) and the motivation to reinstate social contact of birds, rats and mice in open fields (Gallup and Suarez, 1980; Suarez and Gallup, 1981). Therefore, it is unlikely that the differences in jumping behavior between W/W and D/D chicks are due in large part to different motivation for social reinstatement.

Using the righting response test, we can exclude the possibility that the worse performance of W/W chicks in the avoidance test was due to vestibular and motor deficits (Figure 3), but it seems to reflect the level of fear with respect to the lower 5-HTT expression. Chicks of all three 5-HTT genotypes can be regarded to have similar vestibular or motor functions.

Further, it was assumed that depth perception may be related to time of hatching, i.e., older birds are more capable of discriminating and estimating depth more accurately. Nevertheless, our experimental data are not able to support this hypothesis, regarding the explanation for the better performance of D/D chicks in the test because we found no different timing of hatching in our rearing process (data not shown) and no significant difference in mean body weight of newly hatched chicks between the three genotypes (Table 3). Serotonergic axons are also found in the occipital lobe of the macaque monkey (de Lima et al., 1988) and the human (Gerstl et al., 2008), an area which is responsible for the visual orientation, motion and depth perception (Murray et al., 2012; White et al., 2013; Bridge, 2016). Interestingly, several pharmacological studies using the drug 3,4-methylenedioxymethamphetamine (MDMA, ecstasy), which is toxic to central serotonin neurons, have demonstrated that 5-HT plays a pivotal role in the visual orientation processing via lateral inhibition between neighboring orientation-sensitive neurons in the primary visual cortex (Brown et al., 2007; Dickson et al., 2009; Murray et al., 2012; White et al., 2013). In this context, we cannot rule out the possibility that the results obtained with the elevated balance beam represent not only differences in fearfulness, but also differences in the visual orientation and depth perception processing affected by alterations of the downstream 5-HT transmission. It is thus intriguing to investigate this issue in future studies.

**Table 3:** Body weight (g) of newly hatched chicks of three genotypes (W/W, W/D and D/D).

Genotype	Body weight	S.E.	*N*
W/W	40.8	0.37	77
W/D	40.6	0.37	69
D/D	39.7	0.36	73

*One-way ANOVA followed by Tukey HSD post hoc test (P > 0.05)*.

Trying to validate our interpretation of the results from the avoidance test, we performed the arousal test with the same chicks at 5 weeks after hatching, but not at the earlier stages of age for the following reason. In similar open field tests, behavioral responses of chicks have been shown to rapidly change in the post-hatching periods (Heiblum et al., 1998; Balážová and Baranyiová, 2010). For instance, in White-Leghorn chicks the latency to walking decreased sharply after day 1 post-hatching (Heiblum et al., 1998), whereas in broilers another finding was a significant decrease in horizontal locomotor activities between the first and the second week of age, from an about 16-fold higher horizontal locomotor activity at the first week, and no significant difference in open field activities after this time period (Balážová and Baranyiová, 2010). Our time-consuming arousal test due to a large number of chicks took several days and therefore was performed at 5 weeks of age in order to avoid this possible age-related cofounding effect. The test examined the responses to a brief visual isolation but within the home compartment. Importantly, chicks had always acoustic contact to their peers, which has added an important social component to the test situation (Vallortigara, 1988; Vallortigara and Zanforlin, 1988). In general, female chicks react more to such social acoustic stimuli than males (Jones, 1977). Compared to older chicks, 2-day old chicks showed high ambulation latencies in an open field situation in the total absence of any conspecific calls, and the presence of conspecific calls had no effects on their behavior (Vallortigara, 1988). This is probably in these newly hatched birds due to that the isolation from peers is at that early stage perceived as most frightening (Vallortigara, 1988) and the birds are inexperienced. This response changed already at the age of 7 days, where chicks, as one would expect, start moving fastest in the absence of conspecific calling (Vallortigara, 1988). Interestingly, it does not matter whether the peers emit distress calls or trills of pleasure, chicks were shown to react similar to both probably as a result of an unspecific social stimulation (Vallortigara, 1988). Social vocalizing is recognized by chicks and affects their behavior, while a more intense reaction seems to be linked to a higher level of arousal (Vallortigara, 1988). Different to the behavior in a complete novel environment, i.e., a classical open field test (outside the home compartment) where adult D/D hens were shown to be most actively exploring and thus less fearful (Krause et al., 2017), the locomotor activity measured in this study indicated more the arousal in order to return to the peer group, which resulted from visual social separation in the home compartment, but with acoustic (Vallortigara, 1988) and olfactory contact to the flock. The peer group might at this age stage be more important than for older chicken. This resembles findings from several animal studies including mice, rats and chickens, which estimated fear and arousal responses to acute and chronic stresses or to social isolation (Goldsmith et al., 1978; Roth and Katz, 1979; Katz et al., 1981; Heiblum et al., 1998). For young chickens, the vicinity to the social group might provide shelter from e.g., predation and in a natural context at the age of 5 weeks the peer group is also usually linked to maternal presence (Edgar et al., 2013, 2016). Thus, a high level of locomotor activity here reflects likely a high arousal and fear level as these chicks invest more, in terms of locomotion, into trying to return to the peer group. Thus, the high level of locomotor activity of the W/W hens is likely to be strongly affected from the age-dependent social pressure to return to the groups (Faure et al., 1983; Vallortigara, 1988). Also, it was shown that acute exposure to a non-traumatic stress (light and white noise) increased the locomotor activity, in contrast to chronic stresses that reduced the activity (Roth and Katz, 1979; Katz et al., 1981). Interestingly, the same effects were found due to social isolation. Goldsmith et al. (1978) have demonstrated that, following a short duration of individual isolation, isolated mice showed greater locomotor activity, and with longer periods of isolation, they were less active than the group housed conspecifics. Thus, our arousal experiment primarily tested the animal response to acute stress by partial social isolation. Our data showing that W/W chicks, as arousal increased after the short separation from the flock, exhibited higher locomotor activity than D/D chicks were consistent with previous findings that demonstrated that higher 5-HT transmission is associated with an increase in sensory and motoric arousal (Geyer, 1996).

In summary, our data first demonstrated that the avoidance test with an elevated balance beam is capable of measuring different levels of fear in newly hatched birds, and second, that fear-related behaviors are affected by 5-HT genotypes in birds at the very first stage of post-hatch life. Therefore, it is tempting to speculate that the underlying mechanism of these altered behavioral phenotypes might be due to acute 5-HT effects at early post-hatch ages. Furthermore, our results obtained with newly hatched chicks suggest that the species *Gallus gallus* may serve as a suitable model organism in human developmental psychopathology, e.g., of separation anxiety disorder (SAD) that refers to excessive anxiety about separation from home or an attachment figure, which is developed during the childhood and can persist into adulthood (Shear et al., 2006; Ehrenreich et al., 2008), or of infant behavioral inhibition (BI), a fearful temperamental trait which is characterized by strong reactions to environmental and social novelty (Henderson et al., 2015).

## Author Contributions

VPV and LP-V: conception and experimental design and 5-HTT genotyping. VPV, EK and LP-V: avoidance test and arousal test, statistical analysis and data interpretation and writing the manuscript.

## Conflict of Interest Statement

The authors declare that the research was conducted in the absence of any commercial or financial relationships that could be construed as a potential conflict of interest.
